# Immune Response of Adult Sickle Cell Disease Patients after COVID-19 Vaccination: The Experience of a Greek Center

**DOI:** 10.3390/jcm11040937

**Published:** 2022-02-11

**Authors:** Christos Varelas, Eleni Gavriilaki, Ioanna Sakellari, Philippos Klonizakis, Evaggelia-Evdoxia Koravou, Ioanna Christodoulou, Ioulia Mavrikou, Andreas Kourelis, Fani Chatzopoulou, Dimitrios Chatzidimitriou, Tasoula Touloumenidou, Apostolia Papalexandri, Achilles Anagnostopoulos, Efthimia Vlachaki

**Affiliations:** 1 Hematology & BMT Unit, General Hospital “George Papanikolaou”, 57010 Thessaloniki, Greece; varelaschris@gmail.com (C.V.); ioannamarilena@gmail.com (I.S.); evakikor@gmail.com (E.-E.K.); molbiol.gpapanikolaou@n3.syzefxis.gov.gr (I.M.); tasoula.touloumenidou@gmail.com (T.T.); lila.papalexandri@gmail.com (A.P.); achanagh@gmail.com (A.A.); 2 Adult Thalassaemia Unit, 2nd Department of Internal Medicine, Aristotle University of Thessaloniki, Hippokration General Hospital, 54124 Thessaloniki, Greece; philklon@gmail.com (P.K.); g.christod78@gmail.com (I.C.); efivlachaki@yahoo.gr (E.V.); 3 Microbiology Department, Aristotle University of Thessaloniki, 54124 Thessaloniki, Greece; andreaskour@yahoo.gr (A.K.); fchatzop@auth.gr (F.C.); dihi@auth.gr (D.C.)

**Keywords:** sickle cell disease, COVID-19, complement, hemoglobinopathies

## Abstract

Vaccines against severe acute respiratory syndrome coronavirus 2 (SARS-CoV-2) are essential weapons to control the spread of the coronavirus disease-19 (COVID-19) pandemic and protect immunocompromised patients. With a greater susceptibility to infection, sickle cell disease (SCD) patients are considered as “high risk” patients during the current COVID-19 pandemic. In our study, we try to determine the immune response of adult SCD patients monitored at our center after the first and second dose of the qualified mRNA vaccines available and correlate them to several disease-specific markers, as well as complement activation. The results demonstrate that the levels of neutralizing antibodies (nAbs) against SARS-CoV-2 were adequate for most patients studied after the second dose and there seemed to be a certain association with complement activation. Further studies are critical to determine the durability of this immune response and the potential benefit of a third dose.

## 1. Introduction

The constantly evolving threat of the novel coronavirus (SARS-CoV-2) disease-19 pandemic (COVID-19) has put under pressure healthcare systems around the globe and is considered a major concern of public health significance that has already caused millions of deaths [1]. Hemoglobinopathies such as thalassemias and sickle cell disease (SCD) are genetic disorders with a rather high prevalence among humans and are associated with healthcare system overload and the need for intensive and multisystemic follow-up in specialized centers [2]. SCD is characterized by chronic hemolytic anemia, recurrent painful vaso-occlusive events and progressive multiple organ damage. SCD patients suffer from functional hyposplenism and systemic vasculopathy which can often lead to end organ dysfunction and a high risk of thrombosis [3]. Due to this immunocompromised status, they have been included in the “high risk” category of the population during the period of the COVID-19 pandemic. In addition, SCD patients may suffer from both acute and chronic complications which require hospitalization and close contact with the medical system. An overlap has been noticed in clinical manifestations of fever and lung disease in the context of COVID-19 and SCD. The increased complications amplify health care utilization [4,5].

One other key factor in SCD pathophysiology—a pathophysiology much more complicated than originally thought—that places patients in the high-risk category may be complement activation. The role of the innate immune cells in SCD seems to provide a novel field of research, as it was recently reported to a considerable extent by Allali et al. [6]. The innate immune system seems to be a major factor of ongoing inflammatory processes in SCD. Activated innate immune cells are not just deleterious protagonists in SCD; they play a specific role. Their interlinked actions likely result in the main complications of SCD, such as vaso-occlusive crises (VOC), acute chest syndrome (ACS) and stroke. In this context, the complement system provides protection against intruders and regulates host homeostasis. This system functions as a cascade in which inflammatory mediators or opsonins result from the successive cleavage of inactive proteins into their functional fragments. Moreover, it is the alternative complement pathway (AP) that has been mainly reported to be activated in SCD [7]. The quantification of soluble C5b-9 is considered as the most definite biomarker of terminal complement activation in SCD to date and depicts systemic complement dysregulation [8,9].

Despite the lack of specific and effective therapy options for SARS-CoV-2, the introduction of novel vaccines against COVID-19 was deemed the best solution not only for the protection of high-risk patients against devastating complications of the pandemic but also for the development of robust herd immunity and the reduction in hospital admissions, since they were proven to be both safe and efficient [10]. Several clinical trials globally utilized either conventional vaccine platforms that were virus and/or protein-based and were already used in humans, or next generation platforms that could be developed solely on the sequence of the antigenic viral proteins and included viral vector, nucleic-acid-based and antigen-presenting-cell vaccines [11]. After the successful completion of phase III studies, the Greek government decided to enlist two viral-vector-based vaccines (AZD1222, Oxford/AstraZeneca, and Ad26.COV2.S, Janssen) and two mRNA vaccines (mRNA-1273, Moderna, and BNT162, BionTech/Pfizer) in the country’s vaccination campaign. However, other questions arise that concern the ability of the general population to achieve adequate and durable immune response after their vaccination. These questions extend further to patients suffering from hematologic diseases, including adult SCD patients, due to their impaired immunity and to the limited information available on whether and to what extent they could mount functional immune responses, as these patients were generally excluded from vaccination trials [12]. Several assays have been developed to measure neutralizing antibodies (nAbs) against COVID-19, since they are considered a marker of real-time protection [13]. In fact, humoral responses post-SARS-CοV-2 vaccination have been studied in patients with hematologic malignancies, hematopoietic cell transplant (HCT) recipients and/or after CAR-T cell therapy [14,15,16]. However, there are still not enough data on the immune response achieved by adult SCD patients after their COVID-19 vaccination.

Moreover, in a country such as Greece, where there is distrust in the safety, the necessity and the possible rare complications of the vaccination in a large proportion of the population [17], SCD patients are at risk of being misled by populists and “conspiracy theory” fans and may be hesitant in proceeding with their vaccination. This is especially problematic since SCD is a clinical condition characterized by repetitive vaso-occlusive events, a predisposition to infection and a higher risk of thromboembolic disease [18]. 

Given the fact that adult SCD patients are included in the high-risk category of the population and were, subsequently, among the first to qualify for vaccination against COVID-19 and given the lack of relevant data available concerning their immune response after the first and second doses of the vaccine, we studied the levels of protective antibodies against COVID-19 in adult SCD patients after their vaccination.

## 2. Methodology

We prospectively studied adult SCD patients monitored at our center who received at least one dose of one of the qualified available vaccines against COVID-19 until August 2021 and collected their written informed consent to participate in the study. Our study was conducted in accordance with the Declaration of Helsinki. Moreover, our study of complement activation in SCD patients was approved by the Committee for Ethics and Bioethics of Aristotle University of Thessaloniki. Inclusion criteria were age > 18 years, establishment of SCD diagnosis through gel electrophoresis and amplification-refractory mutation system (ARMS) or allele-specific polymerase chain reaction (ASP) and no previous vaccinations against COVID-19. Patient examination during the study’s time points did not reveal major findings. All patients were afebrile and in good clinical condition. It should be noted that, due to Greece’s vaccination campaign at that time, adult SCD patients were eligible for receiving mRNA vaccines only. These were BNT162b2, manufactured by Pfizer–BionTech and mRNA-1273 by ModernaTX, Inc., (Cambridge, MA, USA) and they were approved and recommended by the US Food and Drug Administration (FDA) and the European Medicines Agency (EMA). We used our center’s approved assay to detect nAbs against SARS-CoV-2 in patients’ sera (ELISA, cPass™ SARS-CoV-2 nAbs Detection Kit; GenScript, Piscataway, NJ, USA). 

The cPass™ technology allows the rapid detection of total neutralizing antibodies (nAbs) in a sample to be performed by mimicking the interaction between the virus and the host cell. In order for a virus to infect a host cell, a viral receptor binding protein (RBD) first needs to interact with the host cell’s membrane receptor protein (ACE2). The virus–host interaction and subsequent viral infection of the host cell leads to the activation of an individual’s immune response which generates a population of antibodies against the virus. Some of these antibodies can bind to the virus, but not necessarily block the viral infection. Other antibodies can bind to the RBD in a way that blocks the interaction with the ACE2 receptor. The cPass™ technology helps to distinguish whether a sample contains NAbs that may specifically block the interaction, therefore the viral entry into the host cell. Levels ≥ 30% are considered as positive, while levels of ≥50% are considered as highly protective against severe disease [19]. Samples were collected approximately ten days after the first dose and twenty-one days after the second one. Serum was separated within 4 h from blood collection and stored at −80 °C until the day of measurement.

Renal damage was established as urine protein/24 h ≥ 500 mg in at least 2 different time points. Results were approved from our center’s biochemistry laboratory. Pulmonary hypertension was defined as PASP > 25 mmHg in 2 different echocardiograms with at least a 6-month interval between them. In addition, we tried to determine if there was a possible correlation among patients’ characteristics such as their age, usage of anticoagulants, pulmonary hypertension, previous episodes of vascular strokes, concomitant hydroxycarbamide administration, regular transfusions and renal damage. One other factor studied was the activation of complement in these patients. We were able to collect samples from our patients and measure quantitative results of complement activation before their vaccination against COVID-19. Complement activation was detected in the patient’s sera by measuring soluble human C5b-9 with a commercially available enzyme-linked immunosorbent assay (ELISA) kit (Quidel). The MicroVue SC5b 9 Plus Enzyme Immunoassay measures the concentration of the terminal complement complex (TCC), thereby giving an indication of the status of the terminal complement pathway in the specimen. It uses a monoclonal antibody to the C9 ring of TCC to capture the complex. The trapped TCC is subsequently detected with HRP-conjugated antibodies that bind to antigens of the soluble C5b 9 complex. The samples were tested in duplicates. Our laboratory’s upper normal limit for soluble C5b-9 was defined at 245 ng/mL. Correlation between continuous variables (patient characteristics and immune responses) was assessed with the Pearson or Spearman correlation coefficients. *p*-values ≤ 0.05 were considered significant. Data were analyzed using the statistical program SPSS 23.0 (IBM SPSS Statistics for Windows, Version 23.0; Armonk, NY, USA; IBM Corp). Descriptive statistics are described as median (range) values. The level of statistical significance was defined at 0.05.

## 3. Results

Among 53 adult SCD patients monitored at our center, 25 were studied after their first dose with an mRNA vaccine, of whom 23 were vaccinated with BNT162b2 (Pfizer–BioNTech) and 2 with mRNA-1273 (Moderna). All of them received two doses of the same vaccine type each time. Their median age was 40 years old (range of 19–69). The female-to-male ratio was 16 to 9. Patient population included five patients with the SS SCD genotype and 20 with the S/β, which is the most common in Greece. In total, 13 out of 25 patients had a previous cholecystectomy. Although all SCD patients suffer from functional hyposplenism, 11 had also undergone splenectomy during their childhood. Most of our study’s population (80%) was receiving hydroxycarbamide (dosage of 10–15 mg/kg) on a daily basis. Fifteen patients were on anticoagulant treatment plan at the time of our study, either aspirin or direct oral anticoagulants (DOACs). Two patients suffered from a previous vascular stroke (depicted with magnetic resolution imaging), while none of the patients had performed cardiac echograms with findings suggestive of pulmonary hypertension (PASP ≥ 25 mmHg). Furthermore, 12 patients were receiving regular transfusions during the study and 3 presented with findings of renal damage (urine protein/24 h ≥ 500 mg). Increased complement activation was observed in 14 study subjects before the first dose of the vaccine. Patients’ characteristics are summarized in Table 1.

**Figure 1:**
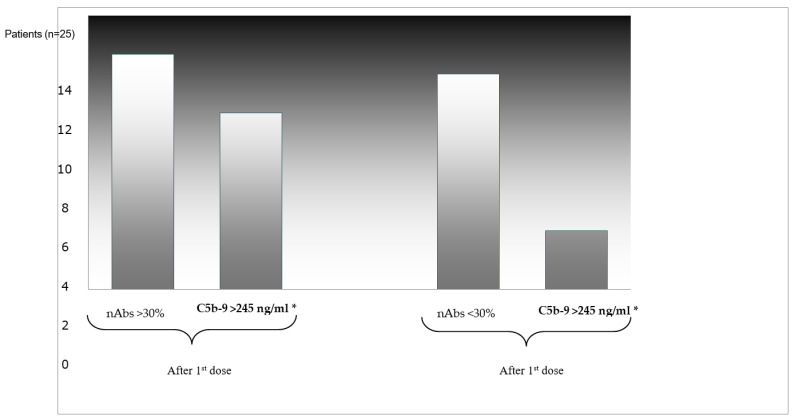
Correlation between levels of nAbs (>30%) and complement activation * (C5b-9 > 245 ng/mL) after the first dose.

Neutralizing nAbs of levels ≥30% were detected in 13 out of 25 patients, 10 of whom (76.9%) had already increased complement activation. On the contrary, among the 12 patients who did not develop neutralizing nAbs after the first dose, only 4 (30%) were found with increased levels of soluble C5b-9 (*p* = 0.022) (Figure 1). In addition, 2 out of 25 patients who had suffered from a vascular stroke in the past did not develop adequate levels of nAbs against SARS-CoV-2. Further correlations between the levels of nAbs and the other markers studied did not establish any statistically significant result.

**Figure 2:**
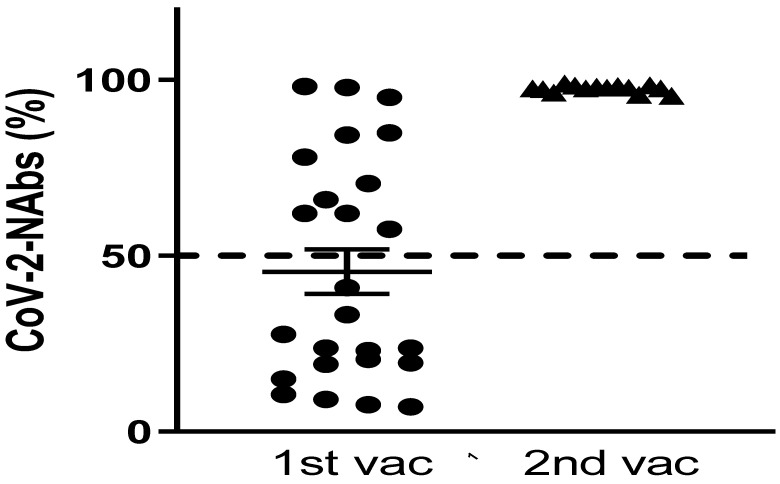
Levels of nAbs after the 1st and 2nd vaccination doses.

After the first dose, only 11 out 25 patients (44%) developed levels of nAbs ≥50% which were associated with clinically important SARS-CoV-2 inhibition and protection against severe disease. Twenty-one days after the second dose, we were able to fully study 15 patients. Results from the remaining 10 patients were not available at the time. All of them developed nAbs with levels of ≥50%, despite the fact that 7 had not achieved adequate levels after the first dose (Figure 2). None of the patients who proceeded to vaccination against COVID-19 reported any complications, including the vaccine-induced immune thrombotic thrombocytopenia (VIITT). Importantly, none of the patients in our study reported previous COVID-19 infection.

## 4. Discussion

This study evaluated the serologic response to SARS-CoV-2 vaccination after a 2-dose regimen—in most patients—of either BNT162b2 or mRNA-1273 COVID-19 vaccine, given 21 days apart, in adult patients with SCD. Neutralizing antibodies are an important component of immunity against SARS-CoV-2 infection, as demonstrated by the efficacy of convalescent plasma in disease attenuation in the general population, as well as immune-compromised populations [20]. Our findings suggest that vaccination against COVID-19 could establish an adequate immune response in adult SCD patients, especially in those with already increased levels of complement activation. Patients with serious SCD-related complications, such as vascular strokes, seemed to develop clinically important levels of nAbs only after the second dose.

Preliminary results from this study demonstrate a rather satisfactory immune response of adult SCD patients after their vaccination against COVID-19. This comes in contrast to results of similar studies in the general context of hematological diseases and immunosuppression. More specifically, recent studies have reported that patients with hematological malignancies, such as chronic lymphocytic leukemia (CLL) and lymphomas, had poor immune response after their 2-dose vaccination against COVID-19, making the vaccines significantly less effective [21,22,23]. However, the underlying causes for poor antibody-mediated immune response to vaccination in these studies’ patients were not only disease-related, but also therapy-related. Many patients had already received anti-CD20 therapy prior to their vaccination, which depletes pools of B cells responsible for antibody production. Higher serum immunoglobin levels at the time of vaccination and lack of active treatment (in the forms of treatment-naïve, off-therapy or ≥12 months from the last cycle of anti-CD20 treatment) had a positive correlation with better response rates. Our study’s patients, although immunocompromised, did not receive any type of immunotherapy and most of them were on a daily schedule of hydroxycarbamide and anticoagulants.

Other studies concerning highly vulnerable patient populations, such as HCT recipients, have dealt with both the B- and the T-cell components of the adaptive immunity in terms of neutralizing antibody viral inhibition and interferon-γ secreting SARS-CoV-2-specific T cells. Their results report active immunosuppression as the major determinant of poor/suboptimal adaptive responses [24]. One possible explanation for this difference is the fact that immunosuppression in SCD is characterized by functional hyposlenism and vasculopathy, whereas, in hematological—especially lymphoid—malignancies by the lack or the dysfunction of B cells responsible for the production of specific antibodies, the use of anti-CD20 monoclonal antibodies, BTK and/or BCL-2 inhibitors further depletes a patient’s B-cell pool.

Despite the fact that our findings suggest a good immune response after vaccination against COVID-19, other questions arise. The discussion mostly revolves around the durability of the protection provided by nAbs against SARS-CoV-2 not only for the immunocompromised patients but also for the general population. Due to SCD’s background of functional hyposplenism, patients around the globe are under different pneumococcal vaccination protocols. The assessment of the immunogenic response of such protocols indicates that anti-pneumococcal immunity may not be optimally maintained, leaving patients vulnerable to invasive pneumococcal disease (IPD) [25]. The obvious hypothesis is that COVID-19 vaccination does not provide long-term optimal immunity in SCD patients in the same pattern with pneumococcal vaccination. This conclusion is amplified by the fact that most countries have already begun an intensive third dose vaccination campaign, not only for the immunocompromised patients but also for the general population.

An important area in the context of our study is the role of complement in the establishment of adequate immunogenic response. The role of complement in the pathophysiology of SCD has not been thoroughly investigated until recently. Although the exact mechanisms that result in complement activation—mainly through the alternative pathway—in SCD are not fully understood, there is solid background to support that complement regulation is a potential target for the prevention of severe complications [7]. Furthermore, it has been reported that complement over-activation in SCD may lead to life-threatening complications that are resistant to standard of care treatment [26]. Nonetheless, patients in our study group with elevated levels of soluble C5b-9 achieved an overall satisfactory immunogenic response while remaining at “steady state” at the same time and without demonstrating signs of complications that could be attributed to complement over-activation.

Limitations of this study include the rather small number of patients enrolled and the fact that our patients received only mRNA-based vaccines. In line with the Greek COVID-19 vaccination campaign, vaccines such as AZD122 manufactured by Oxford/AstraZeneca were not available for SCD patients, due to reports of thrombosis with concomitant thrombocytopenia syndrome following vaccination with these products [27].

Additional data are essential to completely evaluate the duration and efficacy of the two-dose protocol and to elucidate the underlying immune landscape, in order to appropriately select those patients that may fully exploit the benefits of a third anti SARS-CoV-2 vaccination dose. Our study’s patients are currently scheduling their third dose, despite the certain hesitation present among the general population. There is a constant need for newer approaches to vaccinate patients with reduced or rather short-term immunological responses that could include different vaccine design or dosing schedules, as well as combining different coronavirus vaccines. Therefore, SCD patients should continue utilizing protective measures against SARS-CoV-2 (masks, social distancing, etc.) as they are still considered as high risk for severe COVID-19. 

## Figures and Tables

**Table 1:** Patients’ characteristics (*n* = 25).

Age (Median)	40 (19–69)
Sex	F: 16 M: 9
Genotype	ss: 5 s/β: 20
Cholecystectomy	13
Splenectomy	11
Use of anticoagulants	15
Use of hydroxycarbamide	20
Renal damage	3
Transfusions	12
Pulmonary hypertension	0
Previous vascular stroke	2
